# Supplementary material for: Yield Performance of Super Hybrid Rice Grown in Subtropical Environments at a Similar Latitude but Different Altitudes in Southwest China
Source: Plants (Basel). 2025 Feb 21;14(5):660. doi: 10.3390/plants14050660 (PMC11901641; doi:10.3390/plants14050660)
Supplement: Supplementary file 1 [file plants-14-00660-s001.zip › plants-3304475-Supplemental material.pdf]

Supplemental material

Table S1. Growth duration (d), mean maximum temperature (°C), mean minimum temperature and cumulative solar radiation (MJ m^{-2}) of super hybrid rice varieties grown under different plant density in Hanyuan County and Luxian county of Sichuan Province in a field experiment from 2020 to 2021.

Year	Location	Variety	Growth duration (d)		Maximum temperature (°C)		Minimum temperature (°C)		Cumulative solar radiation (MJ m^{-2})	
			TR-HD	HD-MA	TR-HD	HD-MA	TR-HD	HD-MA	TR-HD	HD-MA
2020	Hanyuan	Deyou4727	95	41	27.5	28.3	18.6	20.4	1832.6	677.7
		Luyou727	99	46	27.7	26.8	18.7	19.6	1897.3	720.7
		Nei6you9	97	42	27.6	27.8	18.6	20.2	1854.0	690.4
		Nei6you107	98	43	27.6	27.4	18.6	20.0	1875.9	692.7
		Mean	97	43	27.6	27.6	18.6	20.0	1864.9	695.4
	Luxian	Deyou4727	92	31	28.7	32.9	20.7	25.2	1641.3	544.1
		Luyou727	95	33	28.8	33.2	20.8	25.3	1694.9	583.7
		Nei6you9	93	32	28.7	33.1	20.7	25.2	1657.9	563.4
		Nei6you107	94	32	28.7	33.1	20.7	25.3	1679.2	563.8
		Mean	94	32	28.7	33.1	20.7	25.2	1668.3	563.8
2021	Hanyuan	Deyou4727	95	40	27.6	27.7	18.7	20.1	1841.7	660.3
		Luyou727	98	44	27.9	26.9	18.9	19.8	1911.1	690.4
		Nei6you9	96	41	27.7	27.5	18.8	20.1	1866.4	667.4
		Nei6you107	97	42	27.8	27.3	18.8	20.0	1890.2	652.8
		Mean	97	41	27.7	27.3	18.8	20.0	1877.4	667.7
	Luxian	Deyou4727	91	30	27.3	34.9	20.2	26.3	1527.7	562.0
		Luyou727	94	32	27.4	34.4	20.3	25.7	1574.0	592.8
		Nei6you9	92	31	27.3	35.0	20.2	26.2	1543.3	581.0
		Nei6you107	93	31	27.4	34.8	20.3	26.1	1558.2	580.2
		Mean	93	31	27.4	34.8	20.3	26.1	1550.8	579.0

Note: TR-HD, period from transplanting to heading; HD-MA, period from heading to maturity.

Table S2 Soil properties of the experimental fields

Location	pH	OM (g g^{-1})	TN(g kg^{-1})	TP(g kg^{-1})	TK(g kg^{-1})	AN(mg kg^{-1})	AP(mg kg^{-1})	AK(mg kg^{-1})
Hanyuan	7.6	44.7	2.6	2.2	18.6	158.4	16.1	229.0
Luxian	4.6	28.3	1.6	0.4	16.1	100.2	135.1	130.2

Note: OM, TN, TP, TK, AN, AP and AK are represented the organic matter, total nitrogen, total phosphorus, total potassium, available nitrogen, available phosphorus and available potassium, respectively.

Table S3 Information about the varieties used in this study

Variety	Type	Released year	Female parent	Male parent
Deyou4727	Indica	2013	Dexiang074A	Chenghui727
Luyou727	Indica	2015	Lu006A	Chenghui727
Nei6you9	Indica	2018	Neixiang6A	Luhui9
Nei6you107	Indica	2018	Neixiang6A	Luhui107
